# Sequential Data–Based Patient Similarity Framework for Patient Outcome Prediction: Algorithm Development

**DOI:** 10.2196/30720

**Published:** 2022-01-06

**Authors:** Ni Wang, Muyu Wang, Yang Zhou, Honglei Liu, Lan Wei, Xiaolu Fei, Hui Chen

**Affiliations:** 1 School of Biomedical Engineering Capital Medical University Beijing China; 2 Beijing Advanced Innovation Center for Big Data-based Precision Medicine Capital Medical University Beijing China; 3 Department of Epidemiology and Biostatistics Institute of Basic Medical Sciences Chinese Academy of Medical Sciences, School of Basic Medicine Peking Union Medical College Beijing China; 4 Information Center, Xuanwu Hospital Capital Medical University Beijing China

**Keywords:** patient similarity, electronic medical records, time series, acute myocardial infarction, natural language processing, machine learning, deep learning, outcome prediction, informatics, health data

## Abstract

**Background:**

Sequential information in electronic medical records is valuable and helpful for patient outcome prediction but is rarely used for patient similarity measurement because of its unevenness, irregularity, and heterogeneity.

**Objective:**

We aimed to develop a patient similarity framework for patient outcome prediction that makes use of sequential and cross-sectional information in electronic medical record systems.

**Methods:**

Sequence similarity was calculated from timestamped event sequences using edit distance, and trend similarity was calculated from time series using dynamic time warping and Haar decomposition. We also extracted cross-sectional information, namely, demographic, laboratory test, and radiological report data, for additional similarity calculations. We validated the effectiveness of the framework by constructing k–nearest neighbors classifiers to predict mortality and readmission for acute myocardial infarction patients, using data from (1) a public data set and (2) a private data set, at 3 time points—at admission, on Day 7, and at discharge—to provide early warning patient outcomes. We also constructed state-of-the-art Euclidean-distance k–nearest neighbor, logistic regression, random forest, long short-term memory network, and recurrent neural network models, which were used for comparison.

**Results:**

With all available information during a hospitalization episode, predictive models using the similarity model outperformed baseline models based on both public and private data sets. For mortality predictions, all models except for the logistic regression model showed improved performances over time. There were no such increasing trends in predictive performances for readmission predictions. The random forest and logistic regression models performed best for mortality and readmission predictions, respectively, when using information from the first week after admission.

**Conclusions:**

For patient outcome predictions, the patient similarity framework facilitated sequential similarity calculations for uneven electronic medical record data and helped improve predictive performance.

## Introduction

In recent years, personalized medicine and clinical decision-making support have become popular issues and hot research fields such as modeling with electronic medical records to assist clinicians in diagnosing diseases [1-3], predicting length of hospital stay [4,5], and predicting patient death and other outcomes [4,6-8]. Because electronic medical record data accumulate quickly, sufficient data exist for conducting data-driven studies, big data mining, and constructing predictive models. Using patient similarity measures calculated from electronic medical record data to select study cohorts for building personalized models has improved predictive performances [9,10].

Previous studies [11-14] have demonstrated the effectiveness of personalized predictive models. Wang et al [11,12] used similarity-based models to predict diabetes and liver disease risk. Li et al [13] successfully identified 3 distinct subgroups of type 2 diabetes based on the calculated patient similarity. Wang et al [14] derived a local spline regression-based method for patient embedding and patient similarity measurement to predict cardiovascular disease risk. However, these studies [11-14] merely evaluated patient similarity based on cross-sectional information, rather than using the complete longitudinal information stored in the electronic medical record system. For a hospitalized patient, the longitudinal information represents the clinical trajectory from admission to discharge; it may include a series of clinical events performed on a patient and multiple laboratory tests. Longitudinal data should be better than cross-sectional data in predicting patients’ outcomes due to the rich information on medical behavior and disease progression. Thus, we can assume that longitudinal information in conjunction with patient similarity measurements will further improve outcome prediction, which will facilitate the move toward personalized medicine.

Unfortunately, as is typical of real-world data, electronic medical record data are usually heterogeneous, irregular, and uneven, which presents challenges for modeling and measuring similarity [15]. These problems are more severe for sequential information than they are for cross-sectional information. Thus, many researchers transform longitudinal data into static data. Lee et al [16] extracted various clinical and vital signs during the first 24-hour intensive care unit stay. These longitudinal variables were transformed into static data by calculating the minimum and maximum value for further patient similarity measurement based on the cosine similarity metric. Ng et al [17] used a feature vector representation method to aggregate longitudinal patient data by calculating counts for categorical variables (diagnoses, medications, and procedures) and arithmetic means for numeric lab test data. Sun et al [18] represented 2-hour temporal data for each patient by computing the means and variances or wavelet coefficients.

Because few analyses [15-18] have taken event sequences into consideration for similarity measurements, we aimed to develop a new framework for patient similarity measurement that can make use of cross-sectional information and 2 types of sequential information (series of clinical events and multiple laboratory tests) to predict patient outcomes.

## Methods

### Overview

**Figure 1:**
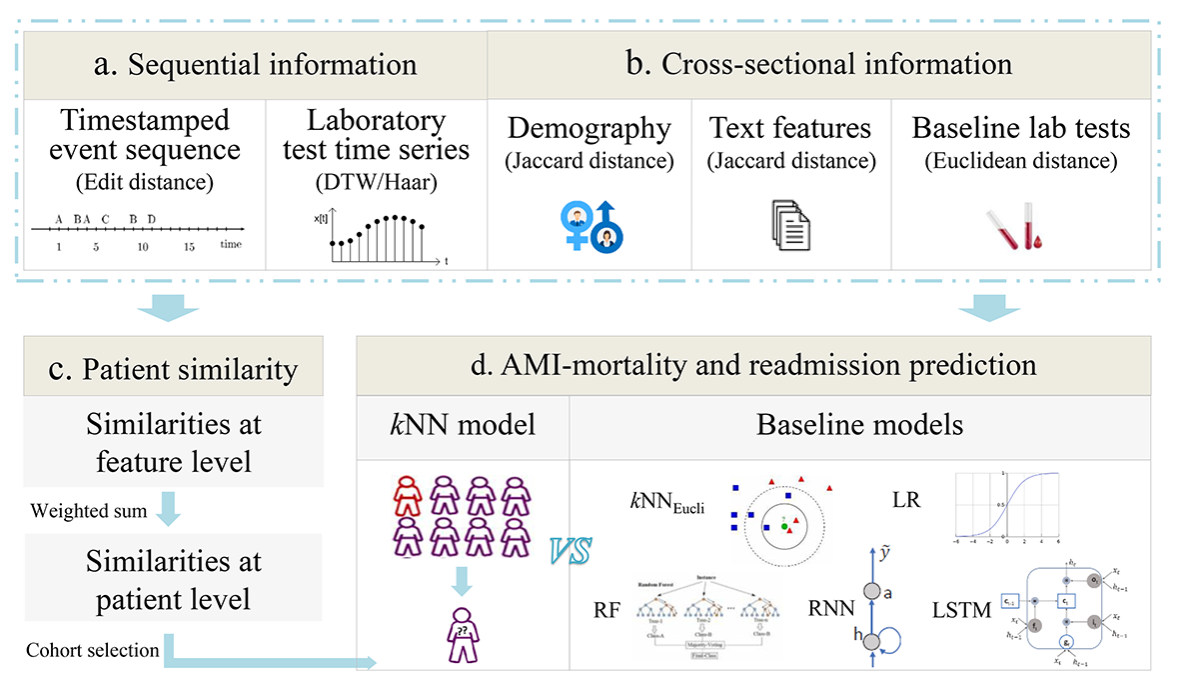
Study workflow: (a) Sequential similarity calculation for timestamped event sequence and time series data, (b) similarity calculation for cross-sectional information, (c) patient similarity measurement based on the weighted sum of similarities calculated in parts a and b, and (d) validation. AMI: acute myocardial infarction; kNN: k-nearest neighbors based on the proposed patient similarity measurement; kNN_Eucli_: k-nearest neighbors based on the Euclidean distance; LR: logistic regression; RF: random forest; RNN: recurrent neutral network; LSTM: long short-term memory network; DTW: dynamic time warping.

In China, the number of patients with acute myocardial infarction is expected to increase from 8 million in 2010 to 23 million in 2030 [19], which usually has a high risk of all-cause in-hospital mortality or readmission, due to unexpected acute myocardial infarction, after discharge. The accurate prediction of these would allow better prognosis and timely intervention. Thus, we focused on the prediction of all-cause in-hospital mortality and unexpected acute myocardial infarction-readmission after discharge of patients with acute myocardial infarction at 3 time points during hospitalization (at admission, on Day 7, and at discharge). Each patient’s clinical trajectory comprised a series of clinical processes (timestamped event sequence) and multiple laboratory tests (time series data) from electronic medical record data. We calculated similarities for both sequential and cross-sectional information and constructed similarity-based models for each time point (Figure 1).

### Similarity for Sequential Information

**Figure 2:**
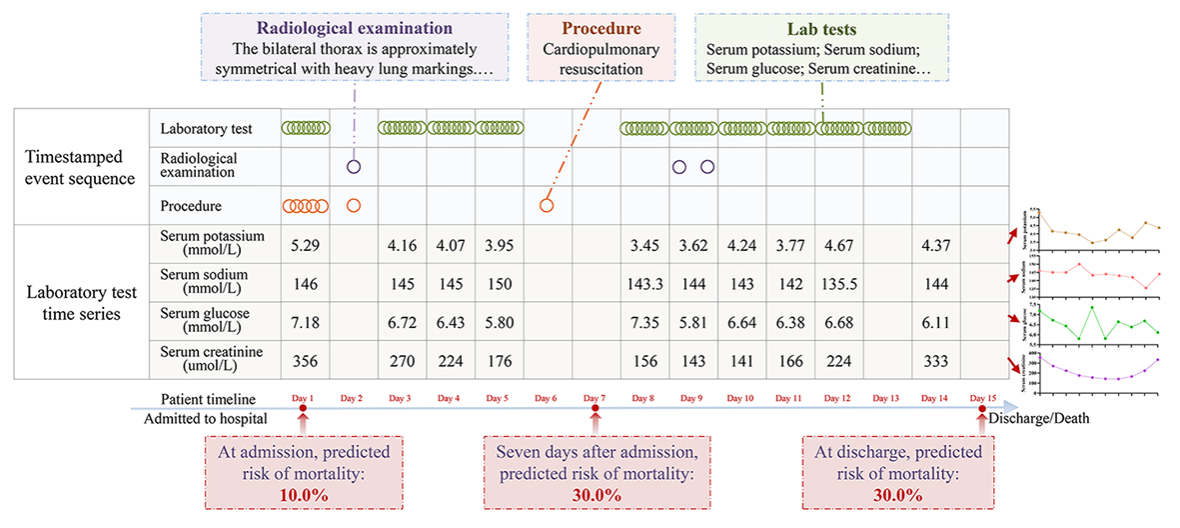
A case study of a patient’s clinical trajectory. All clinical events including laboratory tests, radiological examinations, and procedures are listed sequentially according to patient timeline. The multiple values of each laboratory test comprise the time series data shown in the line chart on the right side of the figure.

Both timestamped event sequence and laboratory test time series data were used to calculate sequential similarity. Laboratory tests data contributed to sequence similarity calculations, and trend similarity calculations from multiple test values, simultaneously. Figure 2 shows an example of a patient’s clinical trajectory.

### Similarity for Event Sequence

An event was a clinical process performed on a patient, such as a serum glucose test, a radiological examination (eg, color sonography), or a procedure (eg, percutaneous coronary intervention). For a series of clinical events, with timestamped information, an event sequence *r* for the patient comprised pairs (*e_i_*, *t_i_*), where *e_i_* was the *i*th clinical event for a patient and *t_i_* was the time point (day) on which the event occurred. Within an event sequence, event e*_i_* was placed before event *e_j_* if *t_i_* was earlier than *t_j_* in the patient timeline. Two events were placed alphabetically if they were performed on the same day.

The edit distance was used to calculate the similarity between 2 event sequences based on how much work was needed to transform one sequence into the other [20,21]. Operations—insertion, deletion, and substitution—were used to change sequence *r_1_* into *r_2_*. For the event–time pair (*e_i_*, *t_i_*) in *r_1_* and (e*_j_*, t*_j_*) in *r_2_*, insertion or deletion were used if *e_i_*≠*e_j_*; otherwise, substitution (ie, changing the occurrence time of an event) was used. We set the edit cost to 1 for insertion and deletion operations, *c*(Ins(*e*))=*c*(Del(*e*))=1, and the cost of substitution was *c*(Sub(*e*,*t_i_*,*t_j_*))=0.5*|*t_i_*–*t_j_*|. Given that we could change sequence *r_1_* to *r_2_* via different series of operations, the operation series with the minimum total cost was taken as the edit distance [21].

For instance, for sequence *r_1_* ={(*A*, 1), (*B*, 2), (*C*, 3), (*D*, 4)} and *r_2_* ={(*A*, 2), (*B*, 5), (*C*, 8) }, where (*A*, 1) indicates that event *A* occurred in the first day after admission, possible operation series could be *Os_1_* = {Del(*A*, 1), Del(*B*, 2), Del(*C*, 3), Del(*D*, 4), Ins(*A*, 2), Ins(*B*, 5), Ins(*C*, 8)}, with a total cost of 7, or *Os_2_* = {Sub(*A*, 1, 2), Sub(*B*, 2, 5), Sub(*C*, 3, 5), Del(*D*, 4)}, with a total cost of 5.5; therefore, the second operation series is optimal. We used a dynamic programming algorithm [20] to solve this minimization problem (Multimedia Appendix 1).

The sequence similarity for a pair of event sequences was



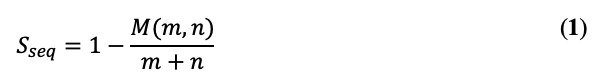



where *M*(*m*,*n*) was the edit distance, and *m* and *n* were the lengths of sequences *r_1_* and *r_2_*. Laboratory test items *S_lab-edit_*, radiological examinations *S_rad-edit_*, and procedures *S_pro-edit_* were represented by 3 individual event sequences.

Time Series Similarity

In the clinical field, a time series can be defined as a consistent, unidirectional change in the value of a biosignal and is, thus, related to the evolution of a patient’s status [22]. In this study, a time series *s* was defined as multiple real values of a laboratory test sorted temporally during a patient’s hospitalization. This type of time series often has different lengths because patients with different diseases have different numbers of laboratory test items. In this situation, the traditional Euclidean or cosine distance was not suitable for calculating the similarity between 2 time series. We used dynamic time warping, which has been frequently implemented to assess similarity between time series data [23,24], to calculate the distance between laboratory test time series. The dynamic time warping algorithm applied dynamic programming algorithm, and the cost for each map was defined by the Euclidean distance between 2 time series (Multimedia Appendix 1). By using the dynamic time warping algorithm, we obtained the optimal alignment and the cumulative distance between 2 time series when mapping one time series onto the other [25].

The trend similarity *S_DTW_* for *s_1_* and *s_2_* was



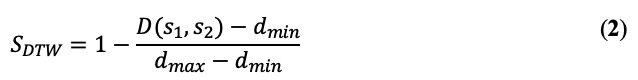



where *D*(s*_1_*,s*_2_*) was the final cumulative distance between *s_1_* and *s_2_*. The minimum and maximum values of all pairwise distances were denoted as *d_min_* and *d_max_*, respectively.

We also used Haar wavelet decomposition method to assess similarity. The Haar wavelet-based method is highly dependent on time series length; therefore, linear interpolation to ensure time series satisfied length requirements. Using discrete Haar wavelet decomposition, each time series was represented by several Haar wavelet bases (Figure S3 in Multimedia Appendix 1), and the coefficients of these bases, which described main characteristics and changing trends in the time series [26], were used to calculate Haar wavelet-based trend similarity *S_Haar_*,



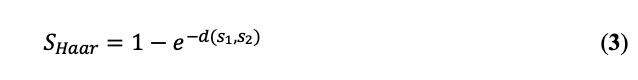



where *d*(*s_1_*, *s_2_*) was the Euclidean distance between 2 groups of coefficients describing *s_1_* and *s_2_*.

The trend similarities between a laboratory test’s multiple test values were calculated using either dynamic time warping or Haar wavelet-based decomposition.

### Similarity for Cross-sectional Information

Cross-sectional information comprised demographic characteristics (age, sex, payment type, and marital status), laboratory tests only performed at admission, and free-text reports of chest x-rays and color sonography.

Demographic characteristics were represented as 0 or 1 in vector *u* based on whether or not the patient was ≥60 years, male, married, and insured (specific medical insurance). To assess demographic feature similarity for patients *i* and *j*, we used Jaccard similarity



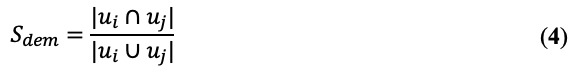



We calculated Euclidean distance–based similarities for laboratory tests performed only at admission. The feature similarity for these cross-sectional laboratory tests (S*_lab_*) was defined as 1 – normalized Euclidean distance, using minimum–maximum normalization.

The free-text reports were in English in the public data set and Chinese in the private data set. For reports written in Chinese, we performed 3 steps to extract features: corpus-of-interest construction, word segmentation, and feature reconstruction (Figure S4 in Multimedia Appendix 1). For reports written in English, we directly identified features of interest (high frequency of occurrence and related to acute myocardial infarction, for example “LVEF,” because patients with high LVEF usually have better cardiac function and prognosis). A text feature variable was set to 1 if patients’ radiological reports contained this feature and 0 otherwise. Finally, each patient had a set of *h* features from text such as “左室射血分数正常 (Left ventricular ejection fraction (LVEF) was normal)” in the private data set and “Overall normal LVEF” in the public data set. We used Jaccard similarity to calculate similarity for extracted text features (*S_text_*).

### Patient Similarity Calculation

The patient similarity score was the weighted sum of feature similarities. We identified dominant features, to which greater weights were assigned, and set weights for the rest of the features to 0. Weights were assigned separately for mortality or readmission risk prediction tasks. The importance of a feature was determined by the predictive performance when using the similarity calculated on this feature to identify nearest neighbors for death or readmission prediction. The greater the performance, the greater the feature importance. Based on the sample set for weight determination, death risk, for example, of an index patient was predicted as the occurring probability of outcomes status among his top *k* nearest neighbors. We selected near neighbors using the similarity of one of the following features in turn: event sequences of laboratory test items, radiological examinations, and procedures; time series of lab tests having multiple testing values; and cross-sectional features.

We identified 3 dominant features, with a majority voting scheme, that had the highest area under receiver operating characteristic curve (AUROC) values. We optimized feature weights *w*_1_, *w*_2_, and *w*_3_, by 0.05 steps, under the constraints *w_1_* + w*_2_* +*w*_3_ = 1 and w*_1_*≥w*_2_*≥w_3_> 0 (Multimedia Appendix 1).

### Predictive Models

#### Similarity–Based Model Configuration

We built several *k*–nearest neighbor classifiers to predict patients’ outcomes based on patient similarity.

We compared predictive performances of *k*–nearest neighbor models built using the sequence similarity alone, the trend similarity alone, and both (Table 1). The subscripts E, D, and H represented the *k*-nearest neighbor model was built by using sequence similarity alone, the dynamic time warping-based trend similarity alone, and Haar decomposition-based trend similarity alone, separately. The subscript ED indicated the *k*-nearest neighbor model was built on both sequence similarity and trend similarity using dynamic time warping, while EH indicated the trend similarity was measured using Haar decomposition.

**Table 1:** The construction of similarity-based predictive models based on different patient similarities.

Similarity used			*k*–nearest neighbor_ED_		*k*–nearest neighbor_EH_		*k*–nearest neighbor_E_		*k*–nearest neighbor_D_		*k*–nearest neighbor_H_
**Sequence similarity**											
	*S*_lab-edit_	Yes		Yes		Yes		No		No	
	*S*_rad-edit_	Yes		Yes		Yes		No		No	
	*S*_pro-edit_	Yes		Yes		Yes		No		No	
**Trend similarity**											
	*S*_DTW_	Yes		No		No		Yes		No	
	*S*_Haar_	No		Yes		No		No		Yes	
Cross-sectional information–based similarity (*S*_dem_, *S*_lab_, *S*_text_)			Yes		Yes		Yes		Yes		Yes

#### Comparison Model Configuration

We compared the predictive performance of each *k*–nearest neighbor model with those of other state-of-the-art predictive models: Euclidean-distance *k*–nearest neighbor, logistic regression, random forest, long short-term memory network, and recurrent neural network models, using either the full set of predictor variables or a set of statistical features, because time series data could not be directly input to Euclidean-distance *k*–nearest neighbor, logistic regression, or random forest models. Cross-sectional information and all flattened time series (padded and concatenated) were input to Euclidean-distance *k*–nearest neighbor, logistic regression, and random forest models, and a set of 6 statistical features for each time series—minimum, maximum, mean, standard deviation, skewness, and time series length—were input to each model with the cross-sectional information. The model with the higher performance for the 2 abovementioned strategies was reported and compared with our similarity-based models (Table S1 in Multimedia Appendix 1).

#### Model Hyperparameters

We searched for the optimal parameters of models by trial and error. Finally, we set *k*=50 for *k*–nearest neighbor and the number of trees to 200 for the random forest model. For the training of logistic regression, long short-term memory network, and recurrent neural network models, we defined loss functions as cross-entropy with an L2-regulation term. The long short-term memory network and recurrent neural network were trained with an adaptive moment estimation optimizer with a sigmoid activation function. For long short-term memory network and recurrent neural network models, the number of units was set to 100, batch size was chosen as 128, and the maximum number of epochs was set to 30. The leave-one-out method was used to evaluate performances of predictive models, with one patient used as a test sample and the rest used for training in each validation round. This method made full use of the validation set and can be used with an imbalanced data set.

Because we aimed to provide an early warning to allow for timely intervention and treatment adjustment, 3 time points, at admission, Day 7, and at discharge were denoted as the index time points. All available information at each index time point was used for determining patient similarity and building predictive models. To ensure robustness, we ran the predictive process 100 times independently and averaged the performances. The differences between models’ performances were considered statistically significant if model A outperformed model B at least 95 times. AUROC and F1-score were used as the main metrics and we also calculated precision, sensitivity, and specificity.

### Data Set and Features

#### Public Data Set

We used the freely accessible critical care database Medical Information Mart for Intensive Care III (MIMIC-III) [27,28]. The MIMIC-III data set was collected between June 2001 and October 2012 from patients admitted to intensive care units at the Beth Israel Deaconess Medical Center in Boston, Massachusetts. It includes patient health information such as demographic data, vital signs, laboratory test results, medications, procedures, diagnosis codes, as well as clinical notes. In this study, we included all records for patients with acute myocardial infarction.

A total of 3010 patients whose primary diagnosis, confirmed with International Classification of Diseases ninth revision codes 410.01 to 410.91, were enrolled in this study. We extracted data on age at admission, sex, payment type, marital status, 42 laboratory tests (23 discrete time series and 19 cross-sectional items), procedures, and radiology reports (34 text features; Table S2 in Multimedia Appendix 1) during hospitalization.

#### Private Data Set

Electronic medical record data used in this study were derived from records of inpatients discharged from a tertiary hospital in Beijing, China between 2014 and 2016. Individual hospitalizations were deidentified and maintained as unique records. Overall, 1846 patients whose primary diagnosis confirmed with the International Classification of Disease, tenth revision, codes I21 and I22 were enrolled. Of the laboratory tests, 103 laboratory tests were used as cross-sectional information (at admission). By Day 7, 27 laboratory tests had 2 or more testing values, and the rest were used as cross-sectional information. At discharge, 63 and 40 laboratory test items were treated as time series and cross-sectional information, respectively. For radiological reports, a set of 36 text features (Table S2 in Multimedia Appendix 1) was obtained.

#### Inclusions and Exclusions

For both data sets, few patients underwent a chest x-ray or a color sonography examination during the first week after admission; therefore, text features were not extracted from radiological reports for further similarity calculation when using information before the first week after admission. The event sequence that comprised radiological examinations was also excluded from sequence similarity calculation because few events occurred at admission. Additionally, a total of 164 patients with a length of stay less than 7 days were excluded from the training sample set when the prediction was made for Day 7. Patients with any length of stay were included in the prediction using patient information during a hospitalization episode. Only 33 and 52 patients in the private data set were readmitted within 30 and 90 days, respectively. Thus, no time requirement was used to identify readmission.

## Results

### General

Table 2 presents characteristics and main outcomes of the study population.

**Table 2:** Basic characteristics of acute myocardial infarction patients in MIMIC-III data set and the private data set.

Characteristic		MIMIC-III data set (n=3010), n (%)	Private data set (n=1846), n (%)
**Demographic**			
	Age ≥60 years	2408 (80.0)	1131 (61.3)
	Male gender	1855 (61.6)	1343 (72.8)
	Married	1583 (52.6)	1815 (98.3)
**Medical Insurance**			
	Urban Employee Basic	N/A^a^	1422 (77.0)
	Medicare	2030 (67.4)	N/A
**Events during a hospital stay, n (numbers of events per patient)**			
	Laboratory test	1,044,886 (347)	349,563 (189)
	Radiological examination	19,171 (6)	5827 (3)
	Procedure	19,630 (7)	13,049 (7)
**Outcomes**			
	Acute myocardial infarction-cause readmission, n (%)	554 (18.4)	100 (5.4)
	All-cause in-hospital mortality, n (%)	245 (8.2)	132 (7.2)
	Length of hospital stay, day, mean (standard deviation)	10.0 (6.24)	11.4 (5.85)

^a^N/A: not applicable.

### Public Data Set

**Figure 3:**
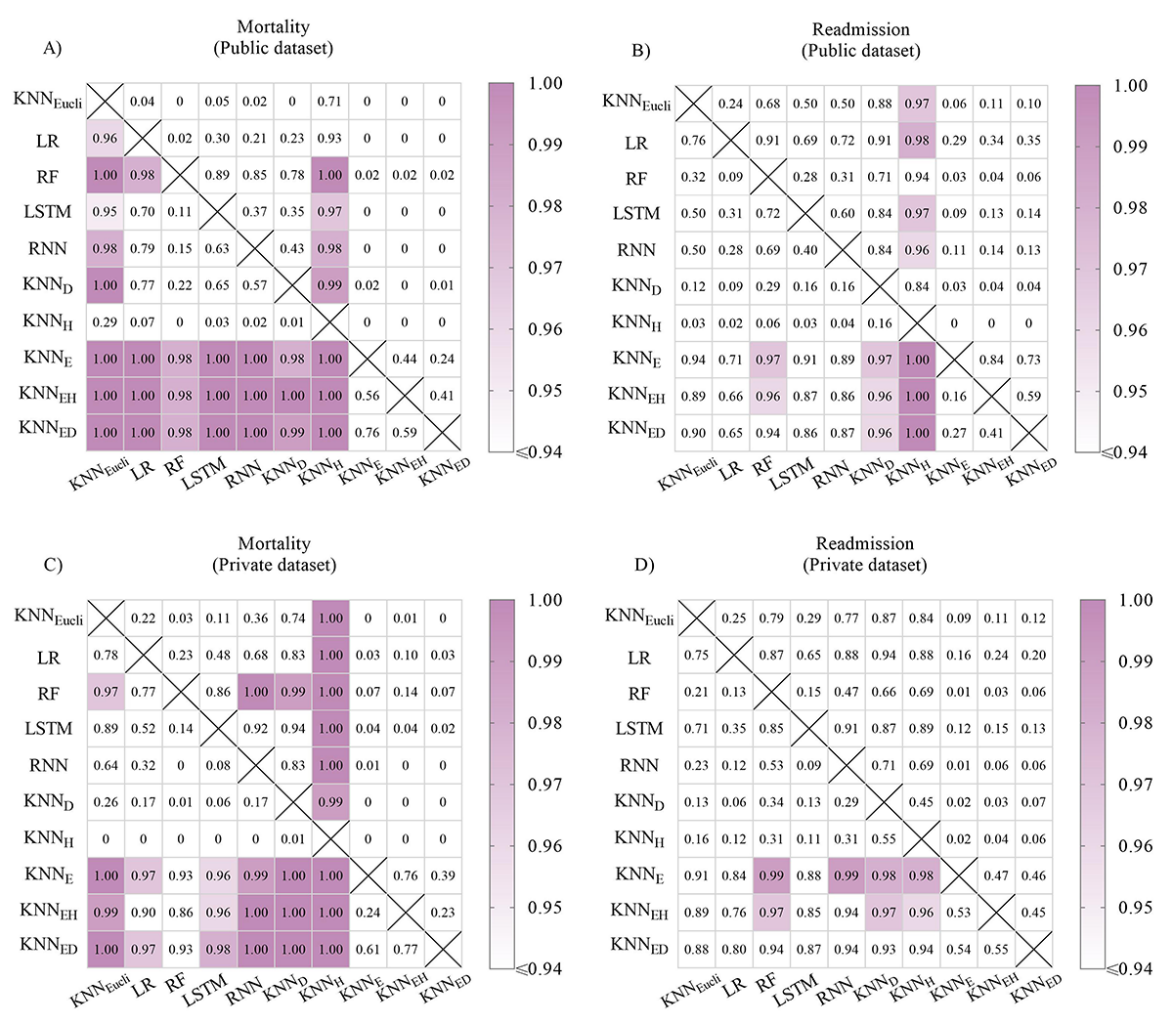
Heatmaps showing the pairwise comparisons among models for predicting mortality (A and C) and readmission (B and D) based on the public (A and B) and private (C and D) dataset. Number in each cell is the percent of times that the model in row had a higher performance than the model in column after 100 experiments. The performance is considered significantly higher if the number is greater than or equal to 0.95, and the corresponding cell is highlighted in color. KNN_Eucli_: Euclidean distance k–nearest neighbor; KNN_D_: kNN built on the dynamic time warping (DTW) -based trend similarity (ie, k–nearest neighbor_D_); KNN_H_: kNN built on the Haar-based trend similarity (ie, k–nearest neighbor_H_); KNN_E_: kNN built on the sequence similarity (ie, k–nearest neighbor_E_); KNN_EH_: kNN built on the sequence similarity and Haar-based trend similarity (ie, k–nearest neighbor_EH_); KNN_ED_: kNN built on the sequence similarity and DTW-based trend similarity (ie, k–nearest neighbor_ED_); LR: logistic regression; RF: random forest; RNN: recurrent neutral network; LSTM: long short-term memory.

When predicting mortality, all *k*–nearest neighbor models built on patient similarities involving events performed best (*k*–nearest neighbor_E_: AUROC 0.878; *k*–nearest neighbor_EH_: AUROC 0.882; and *k*–nearest neighbor_ED_: AUROC 0.883) and significantly outperformed all other models (random forest: *P*=.02; all other models: *P*<.001) (Table 3 and Figure 3A). For predicting acute myocardial infarction-cause readmission, *k*–nearest neighbor_E_, *k*–nearest neighbor_EH_ and *k*–nearest neighbor_ED_ also had the highest AUROC values (Table 3), and the 3 *k*–nearest neighbor models also performed best in mortality and readmission prediction when evaluated with F1-scores. There were no significant differences among *k*–nearest neighbor models involving events for mortality (*k*–nearest neighbor_E_ and *k*–nearest neighbor_EH_: *P*=.44; *k*–nearest neighbor_ED_ and *k*–nearest neighbor_E_: *P*=.24; *k*–nearest neighbor_EH_ and *k*–nearest neighbor_ED_: *P*=.41) and readmission predictions (*k*–nearest neighbor_E_ and *k*–nearest neighbor_EH_: *P*=.84; *k*–nearest neighbor_ED_ and *k*–nearest neighbor_E_: *P*=.73; *k*–nearest neighbor_EH_ and *k*–nearest neighbor_ED_: *P*=.59) (Figure 3).

**Table 3:** The predictive performance of 100 independent rounds of the outcome prediction on the MIMIC-III data set^a^.

Model	Mortality		Readmission	
	AUROC^b^	F1-score	AUROC	F1-score
Euclidean distance *k*–nearest neighbor	0.756 (0.022)	0.280 (0.030)	0.592 (0.019)	0.332 (0.019)
Logistic regression	0.796 (0.024)	0.336 (0.037)	0.608 (0.022)	0.347 (0.019)
Random forest	0.834 (0.015)	0.362 (0.033)	0.579 (0.015)	0.327 (0.020)
Long short-term memory network	0.809 (0.022)	0.356 (0.043)	0.595 (0.020)	0.339 (0.017)
Recurrent neural network	0.814 (0.018)	0.338 (0.039)	0.590 (0.018)	0.337 (0.018)
*k*–nearest neighbor_D_	0.816 (0.023)	0.373 (0.047)	0.566 (0.022)	0.315 (0.027)
*k*–nearest neighbor_H_	0.746 (0.026)	0.295 (0.035)	0.536 (0.026)	0.295 (0.048)
*k*–nearest neighbor_E_	0.878 (0.017)	0.386 (0.041)	0.623 (0.019)	0.350 (0.018)
*k*–nearest neighbor_EH_	0.882 (0.016)	0.401 (0.044)	0.620 (0.018)	0.350 (0.018)
*k*–nearest neighbor_ED_	0.883 (0.015)	0.406 (0.050)	0.620 (0.019)	0.351 (0.019)

^a^Mean: standard deviation.

^b^AUROC: area under the receiver operating characteristic curve.

### Private Data Set

**Figure 4:**
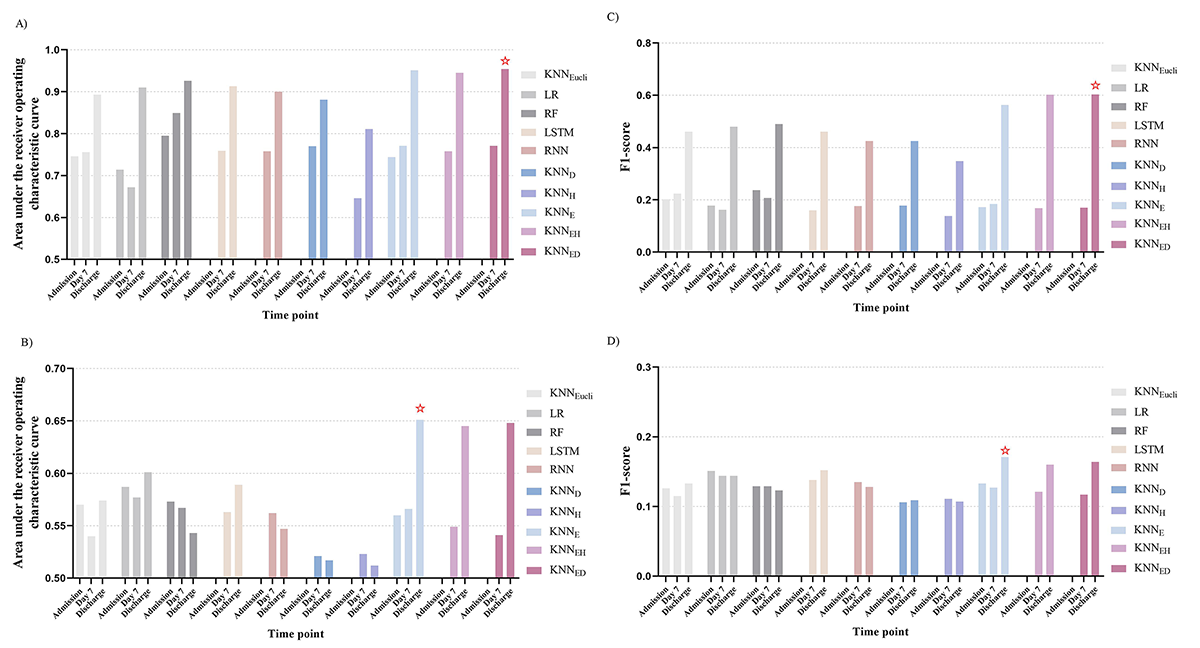
The predictive performance of all models for predicting inpatient mortality (A and C) and readmission (B and D) in terms of area under the receiver operating characteristic curve (A and B) and F1-score (C and D). Stars (☆) indicate the highest predictive performances. No prediction was made at admission for LSTM, RNN, KNN_H_, KNN_D_, KNN_EH_, and KNN_ED_ because of no available temporal information at that time. KNN_Eucli_: Euclidean distance k–nearest neighbor; KNN_D_: kNN built on the dynamic time warping (DTW) -based trend similarity (ie, k–nearest neighbor_D_); KNN_H_: kNN built on the Haar-based trend similarity (ie, k–nearest neighbor_H_); KNN_E_: kNN built on the sequence similarity (ie, k–nearest neighbor_E_); KNN_EH_: kNN built on the sequence similarity and Haar-based trend similarity (ie, k–nearest neighbor_EH_); KNN_ED_: kNN built on the sequence similarity and DTW-based trend similarity (ie, k–nearest neighbor_ED_); LR: logistic regression; RF: random forest; RNN: recurrent neutral network; LSTM: long short-term memory.

When predicting mortality, *k*–nearest neighbor_ED,_ which uses both edit distance–based sequence similarity and dynamic time warping–based trend similarity had the best performance (AUROC 0.954; F1-score 0.603) when using all available information from admission to discharge. It significantly outperformed all other state-of-the-art models (Euclidean distance *k*–nearest neighbor: *P*<.001; recurrent neural network: *P*<.001; logistic regression: *P*=.03; long short-term memory network: *P*=.02) except for random forest (at admission: AUROC 0.795; before Day 7: AUROC 0.849; *P*=.07). (Figure 3C and Figure 4A). Predictive performances of all models improved with time points (at admission, Day 7, and at discharge) except for the logistic regression model (Figure 4A).

For readmission prediction, *k*–nearest neighbor_E_ (AUROC 0.651), *k*–nearest neighbor_EH_ (AUROC 0.645), and *k*–nearest neighbor_ED_ (AUROC 0.648) performed best when using all available information from admission to discharge; however, logistic regression performed best at admission (AUROC 0.589) and before Day 7 (AUROC 0.577) (Figure 4B). The precision, sensitivity, and specificity results of all models are presented in Table S3 (Multimedia Appendix 1).

## Discussion

It is anticipated that predictive modeling based on electronic medical record data will drive personalized medicine and improve health care quality, with many researchers attempting to predict patients’ clinical outcomes, such as death [4,6,7,16,22]; quality of care, such as readmissions [4,7,29,30]; resource utilization, such as length of stay [4,6,31], and diagnoses [6,32]. Patient similarity, calculated based on the electronic medical record data, has improved predictive models’ performances [9,10].

The longitudinal information in electronic medical record data includes timestamped event sequence and laboratory test time series, which are informative and valuable for outcome predictions due to the rich information on medical behavior and disease progression. However, both types of sequential information are usually heterogeneous, irregular, and uneven, presenting large challenges in data preprocessing, feature extraction, and similarity measurement. Therefore, we used 2 strategies to calculate similarity for timestamped event sequence and laboratory test time series separately. The edit distance, which has been widely used to measure distance in analyzing textual strings [33], biological sequences [34], and patient traces [31], was used to calculate similarity for timestamped event sequences.

For time series, 2 main groups of algorithms for similarity calculation can be identified: the time domain algorithm and the transform-based methods [22]. The former worked directly with the raw time series, while the latter reduced original data dimension for further similarity calculation [22]. We used both a time domain (dynamic time warping) and transform-based (Haar wavelet decomposition) to calculate the trend similarity for time series. Dynamic time warping worked better in trend similarity calculations than Haar wavelet decomposition, based on the results for both data sets. Haar wavelet-based trend similarity methods might not be suitable for time series in electronic medical record system. because more information is lost during dimension reduction than that in dynamic time warping. Our findings that dynamic time warping for time-varying features increased predictive performances were similar to those from a previous study [35]. The most frequently selected features were the procedure-based sequence, the serum creatinine level, and the radiological examination-based sequence. This finding inspired us to shed more light on event sequence and specific clinical variables, which helped in identifying similar patients and improving downstream personalized prediction. Generally, dynamic time warping and the edit distance could be used with sequential information having different lengths and helped overcome the challenge of evaluating sequential similarity for uneven electronic medical record data.

Classical time series processing models, such as recurrent neural network and long short-term memory network, could not use event sequence information, and truncation or 0-padding was inevitable in order to process time series with different lengths. Whereas, *k*–nearest neighbor models based on the proposed patient similarity measurement can make use of 2 types of sequential information and performed best in outcome prediction in this study. To the best of our knowledge, this is the first study in which 2 types of sequential information have been integrated and applied to patient similarity measurement. Furthermore, the predictive mechanisms of *k*–nearest neighbor models are more interpretable and transparent for clinicians than some black box models such as random forest, recurrent neural network, and long short-term memory network [16]. In general, our models helped improve predictive performance.

Several prior studies evaluated model performances and compared them with other experiments conducted on the MIMIC-III data set. Zhang et al [4] proposed a fusion model leveraging sequential clinical notes, time series, and static information (AUROC 0.871) that outperformed baseline models for mortality prediction. Guo et al [36] constructed a nomogram to predict in-hospital mortality for myocardial infarction patients (AUROC 0.803). Jiang et al [37] used machine learning to predict in-hospital mortality in sepsis survivors (sepsis: AUROC 0.732; nonsepsis: AUROC 0.830). Suresh et al [38] developed a multitask model (AUROC 0.869) for mortality prediction that outperformed global and separate models. Fan et al [39] predicted in-hospital mortality for acute myocardial infarction patients by building several models such as logistic regression, decision tree, extreme gradient boosting, and random forest; among which, the logistic regression model performed best (AUROC 0.870). In this study, the sequential similarity–based model (AUROC 0.883 for the MIMIC-III data set) had better predictive performance for mortality prediction that those mentioned. The model successfully measured the closeness among patients, helped selecting similar study cohort, and assisted building personalized predictive models. Furthermore, we found that sequence similarity was better at identifying nearest neighbors than trend similarity. This finding coincided with the conclusion that patients’ clinical traces were informative, and similar patient traces might have similar endpoints [31].

Early detection of endpoints for at-risk patients is key for understanding and improving outcomes [5]. In our study, we selected 3 timepoints during hospitalization: at admission, Day 7, and at discharge. At each timepoint, all available data including sequential information were used to predict the outcomes of patients with acute myocardial infarction. For predicting mortality, the performances of all predictive models, except logistic regression, improved with the 3 timepoints. This finding indicated that sequential data helped improve performances of models. The more sequential information involved, the better the predictive performance. This finding verified our initial assumption that longitudinal information in conjunction with patient similarity measurement would facilitate more accurate outcome prediction.

For predicting unplanned readmission, our model performed best on both data sets when all data, from during the whole hospitalization period, were used. This finding sufficiently indicated that patient similarity could significantly boost the performance of readmission prediction. However, unsatisfactory predictive results for readmission prediction were found in our study and have also been found in other studies [4,6]. The reason might be that the readmission condition was multifactorial and complex, such as related to patient medical insurance, economic conditions, and individual factors, thus, it is challenging to make a prediction [4]. In addition, we noted that the performances of all models for mortality and readmission prediction at admission and at Day 7 were significantly lower than those at discharge, possibly because information that is a long temporal interval from discharge was not useful in outcome prediction.

This study had some limitations. First, trend similarity can also be calculated based on time series in the form of abnormality status. This method would require validation in the future. Second, the patient information used in this study was insufficient. The electrocardiogram captures vital signs for patients with acute myocardial infarction and a type of longitudinal information enabling temporal similarity calculation. However, this information was unavailable for the private data set. Therefore, electrocardiograms should be collected and used for similarity measurement in further study.

In this study, we proposed a complete framework for measuring patient similarity that used both sequential and cross-sectional information. The method successfully evaluated sequential similarity, helped deal with the challenge of similarity calculation for uneven electronic medical record data, and improved the performance of predicting patients’ outcomes.

## Appendix

Multimedia Appendix 1 Supplementary material.

## Abbreviations

AUROC: area under the receiver operating characteristic curve
MIMIC-III: Medical Information Mart for Intensive Care III
