# Supplementary material for: Sequential Data–Based Patient Similarity Framework for Patient Outcome Prediction: Algorithm Development
Source: J Med Internet Res. 2022 Jan 6;24(1):e30720. doi: 10.2196/30720 (PMC8778569; doi:10.2196/30720)
Supplement: Multimedia Appendix 1 [file jmir_v24i1e30720_app1.docx]

# Multimedia Appendix 1

## Dynamic programming

A dynamic programming (DP) algorithm[1, 2] was used to obtain the optimal operation series when transforming one sequence into another. For example, for sequence *r*_1_ ={ (*A*, 1), (*B*, 2), (*C*, 3), (*D*, 4)} and *r*_2_ ={ (*A*, 2), (*B*, 5), (*C*, 8) }, we firstly constructed a matrix M $\in R^{(m+1)\times(n+1)},$ where *m*, *n* was the length of *r*_1_, *r*_2_. The value of each cell $M(i,j)$ was the cumulative distance of transforming the first *i* elements of *r*_1_ to the first *j* elements of *r*_2_ using insertion, deletion and substitution. The $M(i,j)$ was calculated with a recurrence relation[1, 2] (Fig. S1).

| $M(i,j)=\left\{ \begin{aligned} max(i,j) if min(i,j)=0, \\ min\left\{ \begin{aligned} M\left( i-1,j \right)+w(e_{i},t_{i}) \\ M\left( i,j-1 \right)+w(e_{j},t_{j}) \\ M\left( i-1,j-1 \right)+K\left( i,j \right) \end{aligned} \right. \end{aligned} \right.$ | (1) |
| --- | --- |

where $w(e_{i},t_{i})$and $w(e_{j},t_{j})$ was the cost of inserting/deleting an event-time pair (defined as 1 in this study). And the $K\left( i,j \right)$ was defined as:

$$K\left( i,j \right)=\left\{ \begin{aligned} 0.5*\left| t_{i} - t_{j} \right| if e_{i}=e_{j} \\ w(e_{i},t_{i})+w(e_{j},t_{j}) if e_{i}\neq e_{j} \end{aligned} \right.$$

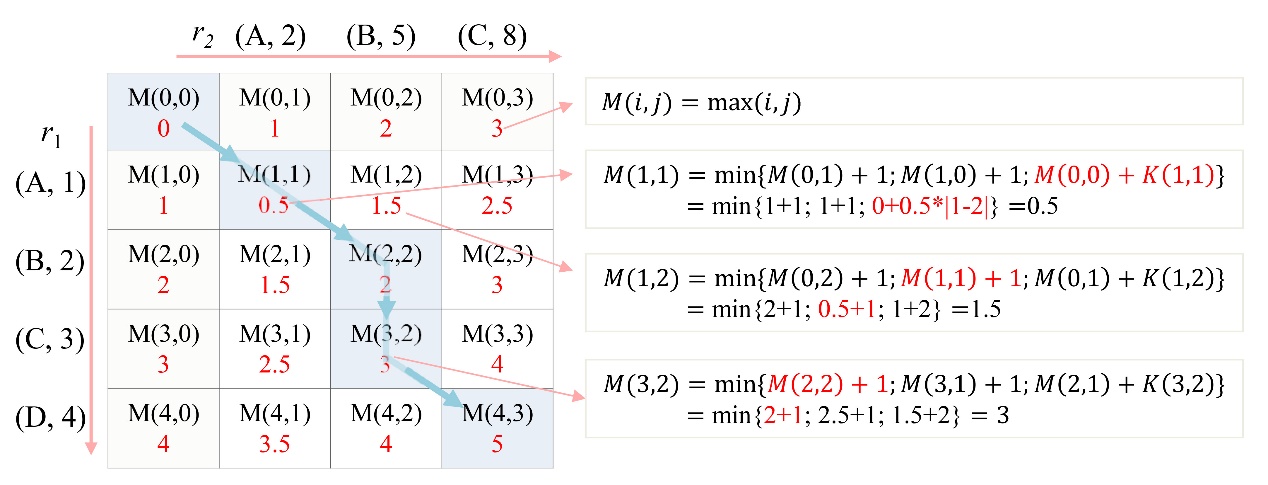


Fig S1. The edit distance calculation for timestamped event sequences based on the dynamic programming algorithm.

## Dynamic time warping

A dynamic time warping (DTW) algorithm[3] was used to obtain the optimal alignment between two time series. For example, for signal *S*_1_ ={3.73, 3.98, 4.36, 4.31} and *S*_2_ ={3.61, 3.71, 4.03, 4.35, 4.26}, we firstly constructed a matrix *d* $\in R^{m\times n},$ where *m*, *n* was the length of *S*_1_, *S*_2_. The value of each cell $d(i,j)$ was the Euclidean distance between the *i* element of *S*_1_ and the *j* element of *S*_2_. Then we calculated the cumulative distance of mapping the first *i* elements of *S*_1_ onto the first *j* elements of *S*_2_ on the basis of a recurrence relation. Fig. S2 visualized the optimal alignment of a pair of signals identified by the DTW algorithm.

| $D\left( i,j \right)= min\left\{ \begin{aligned} D(i-1,j)+d(i,j) \\ D(i-1,j-1)+d (i,j) \\ D(i,j-1)+d(i,j) \end{aligned} \right.$ | (2) |
| --- | --- |


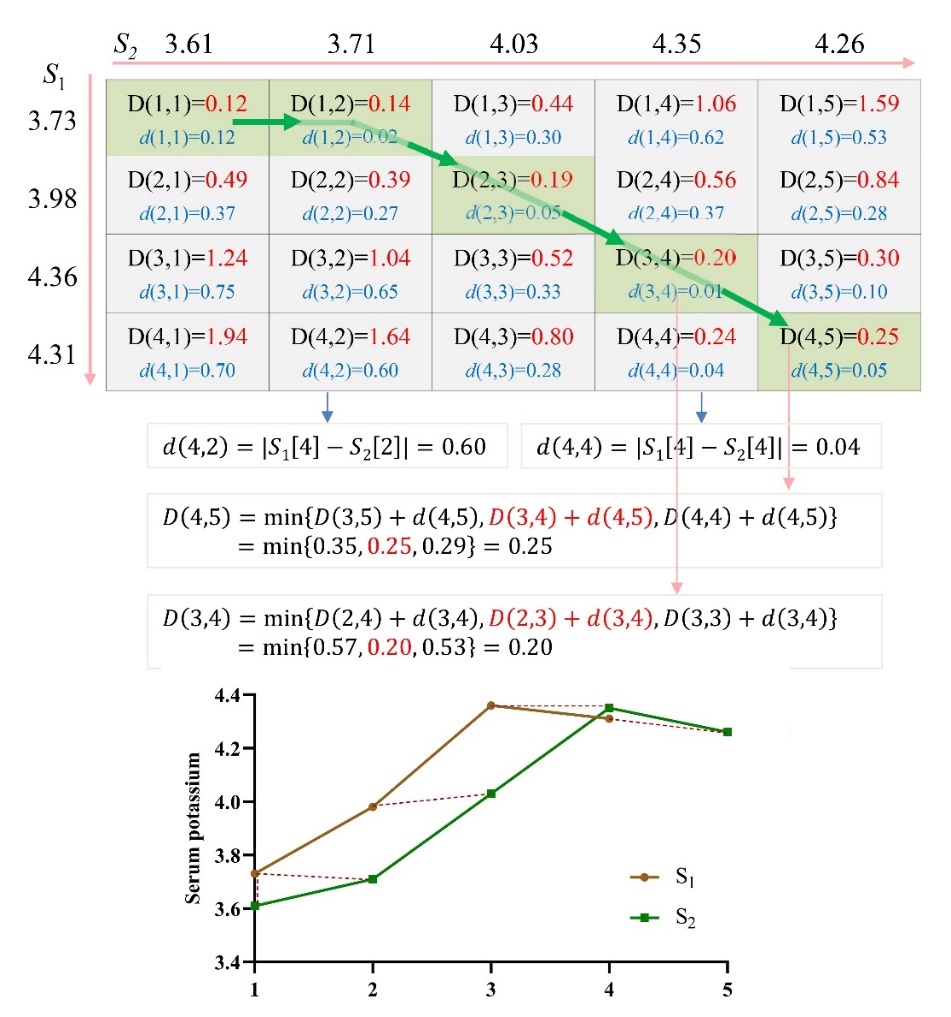


Fig S2. The distance calculation for time series based on the dynamic time warping algorithm.

## Haar wavelet decomposition

To eliminate vertical offsets, each signal was first modified as *S*(*x*)= *S*(*x*) −$\overline{S}$, where $\overline{S}$ is the mean value of the signal *S*. For a discrete laboratory test signal, its Haar series expansion was defined as[4]:

| $f(x)=\frac{1}{\sqrt{N}}[T_{\varphi(0,0)}\varphi_{(0,0)}(x)+\sum_{j=0}^{J-1} \sum_{k=0}^{2^{j}-1} T_{\psi(j,k)}\psi_{(j,k)}(x)]$ | (3) |
| --- | --- |

where *x* is the time point and N was the length of this signal, and *J* was the expansion space (set to 2 in this study). And the $T_{\varphi(0,0)}$, $T_{\psi(j,k)}$ was the coefficients of the father wavelet function $\varphi_{0,0}(x)$ and mother wavelet function $\psi_{0,0}(x)$, separately. The wavelet functions and corresponding coefficients were defined in the Eq(4-1) to Eq(4-5).

| $\varphi_{0,0}\left( x \right)=\left\{ \begin{aligned} 1 if 0\leq x\leq N \\ 0 otherwise \end{aligned} \right.$ | (4-1) |
| --- | --- |
| $\psi_{0,0}\left( x \right)=\left\{ \begin{aligned} 1 if 0\leq x<N/2 \\ -1 if N/2<x\leq N \\ 0 otherwise \end{aligned} \right.$ | (4-2) |
| $\psi_{j,k}\left( x \right)=2^{j/2}\psi(2^{j}x-k)$ | (4-3) |
| $T_{\varphi(0,0)}=\frac{1}{\sqrt{N}}\sum_{x=0}^{N-1} f(x)\varphi_{0,0}(x)$ | (4-4) |
| $T_{\psi(j,k)}=\frac{1}{\sqrt{N}}\sum_{x=0}^{N-1} f(x)\psi_{j,k}(x)$ | (4-5) |

Thus, there were overall four coefficients $T_{\varphi(0,0)}$, $T_{\psi(0,0)}$, $T_{\psi(1,0)}$, and $T_{\psi(1,1)}$ when the expansion space *J* was set to 2. The Haar wavelet bases $\varphi_{0,0}\left( x \right)$, $\psi_{0,0}\left( x \right)$, $\psi_{1,0}\left( x \right)$, and $\psi_{1,1}\left( x \right)$ was visualized in the Fig. S3A. The four bases’ coefficients were identified for each signal and used to calculate Euclidean distance-based pairwise similarities. As shown in Fig. S3B, the *S*_1_’s Haar series expansion remained main characteristics and had similar changing trends with the raw signal *S*_1_, validating that the Haar wavelet-based algorithm could extract signal information efficiently. Furthermore, the identified nearest neighbor according to the Haar wavelet-based similarity were similar to the target signal, which demonstrated the effectiveness of the Haar wavelet-based trend similarity calculation adopted in this study.


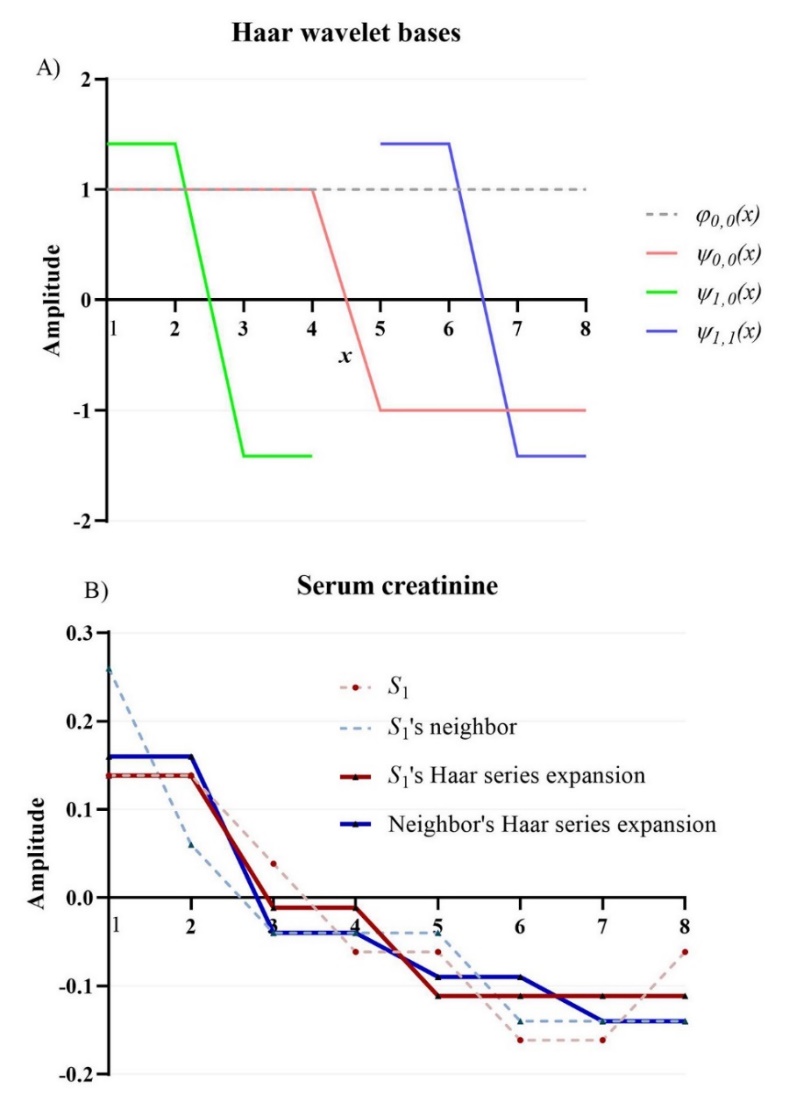


Fig S3. The visualization of a pair of similar temporal signals identified by the calculated Haar wavelet-based trend similarity. A) Four Haar wavelet bases. B) The temporal signal consisted of serum creatinine’s multiple testing values. The longitudinal axis *Amplitude* represents the modified testing value of serum creatinine.

**References**

1. Moen P: **Attribute, Event Sequence, and Event Type Similarity Notions for Data Mining**. 2000.

2. Mannila H, Moen PJICoDW, Discovery K: **Similarity between Event Types in Sequences**. 1999.

3. Giorgino: **Computing and Visualizing Dynamic Time Warping Alignments in R: The dtw Package.** *J Stat Soft* 2009, **31**(9966):402-402.

4. Stankovi RS, Falkowski BJ: **The Haar wavelet transform: its status and achievements**. *Computers & Electrical Engineering* 2003, **29**(1):25-44.

## Text feature extraction


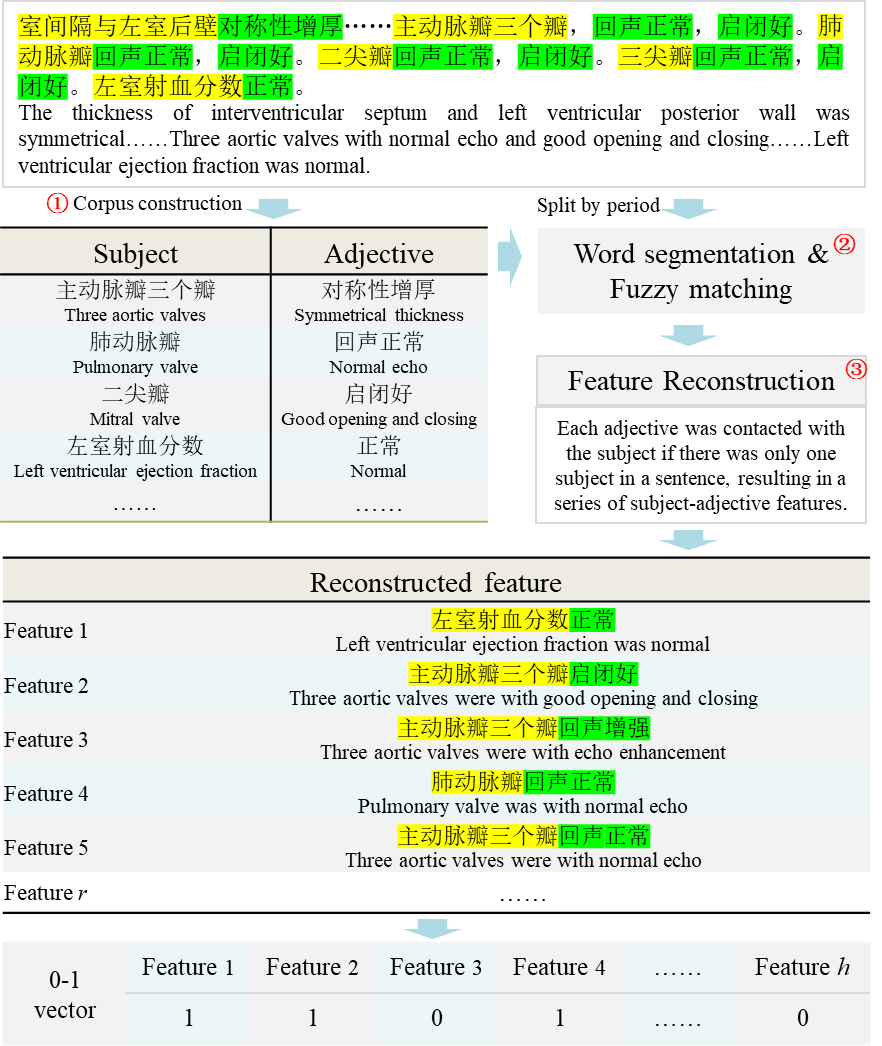


Fig S4. The main steps of text feature extraction and construction based on radiological reports in the private dataset.

## Algorithm 1

| **Algorithm** 1 Patient similarity calculation |
| --- |
| **Input**: training set, testing set |
| 1: FS, FS_t_ ← Calculate feature similarities for training/testing set |
| 2: **for** i = 1 to number of feature similarities by 1 |
| 3: y_pred ← Predict based on the *i*-th FS |
| 4: AUC ← Calculate AUC for y_pred |
| 5: **end** |
| 6: F_1_, F_2_, F_3_ ← Select 3 feature similarities from FS |
| 7: **while** w_1_, w_2_, w_3_ satisfy constrains in Eq(5) **do** |
| 8: PS ←F_1_*w_1_ + F_2_*w_2_ + F_3_*w_3_ |
| 9: y_pred_2_ ← Predict based on the PS |
| 10: AUC_2_ ← Calculate AUC for y_pred_2_ |
| 11: **end while** |
| 12: w_1_, w_2_, w_3_ ←Select scheme with the highest AUC_2_ |
| 14: F_1t_, F_2t_, F_3t_ ←Select accordingly 3 feature similarities from FS_t_ |
| 15: Patient Similarity ← F_1t_*w_1_ + F_2t_*w_2_ + F_3t_*w_3_ |
| 16: **return** Patient Similarity |

## Features for baseline models

Table S1 The total number of features for baseline models

|  | Private dataset | | | Public dataset |
| --- | --- | --- | --- | --- |
|  | At admission | Day 7 | At discharge |  |
| Demographics | 4 | 4 | 4 | 4 |
| Baseline lab tests | 103 | 76 | 40 | 19 |
| Text features | 0 | 0 | 36 | 34 |
| Flattened temporal signals | 0 | 541 (27) | 1181 (63) | 1084 (23) |
| Total (strategy 1)^#^ | 4+103  =107 | 4+76+541=621 | 4+40+36+1181=1261 | 4+19+34+1084=1141 |
| Hand-engineered features | 0 | 27*6=162 | 63*6=378 | 23*6=138 |
| Total (strategy 2)^#^ | 4+103  =107 | 4+76+162=242 | 4+40+36+378=458 | 4+19+34+138=195 |

^#^ We adopted two strategies for processing time series data by using either the full set of predictor variables or the hand-engineered features.

## Text features

Table S2 Extracted text features based on the public and private dataset

|  | Public dataset | Private dataset |
| --- | --- | --- |
| 1 | No pericardial effusion | 左室射血分数正常  (Left ventricular ejection fraction was normal) |
| 2 | Mildly thickened mitral valve leaflets | 左房左室扩大  (Left atrial left ventricular enlargement) |
| 3 | Normal RV chamber size and free wall motion | 主动脉瓣三个瓣启闭好  (Three aortic valves were with good opening and closing) |
| 4 | Normal tricuspid valve leaflets | 主动脉瓣三个瓣开放好  (Three aortic valves were with good opening) |
| 5 | The mitral valve leaflets are mildly thickened | 主动脉瓣三个瓣回声正常  (Three aortic valves were with normal echo) |
| 6 | Mildly thickened aortic valve leaflets | 主动脉瓣三个瓣回声增强  (Three aortic valves were with echo enhancement) |
| 7 | Normal LV wall thickness | 主动脉瓣三个瓣闭合不良  (Three aortic valves were with bad closing) |
| 8 | Regional LV wall motion abnormalities | 右心房室内径正常  (The right atrial ventricular diameter was normal) |
| 9 | Normal pulmonic valve leaflet | 双室流出道内径正常  (The inner diameter of double chamber outflow tract is normal) |
| 10 | Normal aortic diameter | 室间隔与左室后壁开放好  (The ventricular septum and left ventricular posterior wall were well opened) |
| 11 | The left atrium is mildly dilated | 室间隔与左室后壁回声正常  (The echo of ventricular septum and left ventricular posterior wall was normal) |
| 12 | Normal RA size | 室间隔与左室后壁厚径正常  (The thickness of ventricular septum and left ventricular posterior wall were normal) |
| 13 | Mild symmetric LVH | 室间隔与左室后壁厚度正常  (The thickness of ventricular septum and left ventricular posterior wall was normal) |
| 14 | Normal LV cavity size | 室间隔与左室后壁对称性增厚  (Symmetrical thickening of ventricular septum and left ventricular posterior wall) |
| 15 | The aortic valve leaflets are mildly thickened | 室间隔与左室后壁闭合不良  (Poor closure of ventricular septum and left ventricular posterior wall) |
| 16 | Normal mitral valve leaflets | 三尖瓣启闭好  (Tricuspid valve open and close well) |
| 17 | Mildly dilated RA | 三尖瓣开放好  (The tricuspid valve is open well) |
| 18 | Normal aortic valve leaflets | 三尖瓣回声正常  (The echo of tricuspid valve is normal) |
| 19 | Mildly thickened tricuspid valve leaflets | 三尖瓣闭合不良  (Tricuspid regurgitation) |
| 20 | Right ventricular chamber size and free wall motion are normal | 其余各房室内径正常  (The indoor diameter of other rooms is normal) |
| 21 | Normal LA size | 各房室内径正常  (The indoor diameter of each room is normal) |
| 22 | Moderate cardiomegaly | 肺动脉瓣启闭好  (Pulmonary valve was with good opening and closing) |
| 23 | Heart size is normal | 肺动脉瓣回声正常  (Pulmonary valve was with normal echo) |
| 24 | Mild cardiomegaly | 二尖瓣启闭好  (Good mitral valve opening and closing) |
| 25 | The aortic root is normal in diameter | 二尖瓣回声正常  Mitral echo is normal) |
| 26 | Mediastinal contours are stable | 二尖瓣后叶启闭好  (The posterior mitral valve opens and closes well) |
| 27 | The right atrium is normal in size | 二尖瓣后叶回声增强  (Echo enhancement of posterior mitral lobe) |
| 28 | Overall normal LVEF | 纵隔随体位左移  (Mediastinum moves left with body position) |
| 29 | Heart is enlarged | 心影形态未见异常  (The shape of heart shadow is normal) |
| 30 | Atheroma in the descending thoracic aorta | 肋膈角锐利  (Sharp costophrenic angle) |
| 31 | Aorta is tortuous | 双侧胸廓大致对称  (Bilateral thorax is roughly symmetrical) |
| 32 | Pulmonary hypertension | 纵隔无增宽表现  (Mediastinum did not widen) |
| 33 | Atheroma in the aortic arch | 纵隔位置居中无移位  (Mediastinum is centered without displacement) |
| 34 | The tricuspid valve leaflets are normal | 心影增大  (Enlargement of heart shadow) |
| 35 |  | 双侧膈肌光整  (Bilateral diaphragmatic smoothing) |
| 36 |  | 肋膈角欠锐利  (Costophrenic angle is not sharp) |

## Predictive performance

Table S3 The predictive performance of 100 independent rounds of the outcome prediction on two datasets (mean±standard deviation)

| Model | Mortality | | | Readmission | | |
| --- | --- | --- | --- | --- | --- | --- |
|  | Precision | Sensitivity | Specificity | Precision | Sensitivity | Specificity |
| Private dataset (at admission) | | | | | | |
| ${KNN}_{Eucli}$ | 0.172 ± 0.036 | 0.633 ± 0.121 | 0.747 ± 0.093 | 0.072 ± 0.016 | 0.611 ± 0.197 | 0.531 ± 0.192 |
| LR | 0.149 ± 0.027 | 0.746 ± 0.096 | 0.657 ± 0.079 | **0.086 ± 0.018** | 0.555 ± 0.182 | **0.640 ± 0.166** |
| RF | **0.280 ± 0.062** | **0.771 ± 0.059** | **0.835 ± 0.053** | 0.074 ± 0.020 | **0.675 ± 0.172** | 0.491 ± 0.170 |
| ${KNN}_{E}$ | 0.208 ± 0.067 | 0.689 ± 0.120 | 0.761 ± 0.118 | 0.082 ± 0.024 | 0.541 ± 0.217 | 0.608 ± 0.212 |
| Private dataset (Day 7) | | | | | | |
| ${KNN}_{Eucli}$ | 0.296 ± 0.066 | 0.751 ± 0.059 | 0.850 ± 0.052 | 0.072 ± 0.020 | 0.469 ± 0.241 | 0.629 ± 0.224 |
| LR | 0.276 ± 0.059 | 0.664 ± 0.073 | 0.856 ± 0.049 | 0.084 ± 0.020 | 0.537 ± 0.194 | **0.637± 0.183** |
| RF | 0.317 ± 0.060 | **0.847 ± 0.044** | 0.851 ± 0.041 | 0.072 ± 0.012 | **0.691 ± 0.137** | 0.477 ± 0.145 |
| LSTM | 0.235 ± 0.064 | 0.767 ± 0.074 | 0.787 ± 0.077 | **0.089 ± 0.043** | 0.523 ± 0.223 | 0.636 ± 0.219 |
| RNN | 0.226 ± 0.044 | 0.772 ± 0.061 | 0.785 ± 0.056 | 0.079 ± 0.018 | 0.555 ± 0.175 | 0.610 ± 0.168 |
| ${KNN}_{D}$ | 0.262 ± 0.055 | 0.758 ± 0.061 | 0.823 ± 0.056 | 0.069 ± 0.032 | 0.473 ± 0.296 | 0.610 ± 0.283 |
| ${KNN}_{H}$ | 0.270 ± 0.071 | 0.617 ± 0.088 | 0.850 ± 0.085 | 0.069 ± 0.025 | 0.499 ± 0.277 | 0.592 ± 0.275 |
| ${KNN}_{E}$ | 0.360 ± 0.091 | 0.791 ± 0.056 | 0.879 ± 0.047 | 0.075 ± 0.017 | 0.594 ± 0.231 | 0.553 ± 0.218 |
| ${KNN}_{EH}$ | **0.362 ± 0.094** | 0.785 ± 0.062 | **0.880 ± 0.049** | 0.070 ± 0.020 | 0.569 ± 0.240 | 0.555 ± 0.242 |
| ${KNN}_{ED}$ | 0.352 ± 0.073 | 0.808 ± 0.056 | 0.875 ± 0.044 | 0.073 ± 0.028 | 0.567 ± 0.285 | 0.549 ± 0.276 |
| Private dataset (At discharge) | | | | | | |
| ${KNN}_{Eucli}$ | 0.331 ± 0.071 | 0.798 ± 0.061 | 0.864 ± 0.053 | 0.083 ± 0.024 | 0.506 ± 0.207 | 0.640 ± 0.199 |
| LR | 0.341 ± 0.076 | 0.832 ± 0.058 | 0.866 ± 0.045 | 0.085 ± 0.021 | 0.624 ± 0.186 | 0.585 ± 0.177 |
| RF | 0.349 ± 0.074 | 0.856 ± 0.045 | 0.868 ± 0.044 | 0.071 ± 0.016 | 0.577 ± 0.205 | 0.548 ± 0.200 |
| LSTM | 0.320 ± 0.057 | 0.850 ± 0.050 | 0.853 ± 0.041 | 0.094 ± 0.028 | 0.519 ± 0.167 | 0.683 ± 0.155 |
| RNN | 0.291 ± 0.071 | 0.843 ± 0.063 | 0.827 ± 0.061 | 0.074 ± 0.013 | 0.570 ± 0.176 | 0.572 ± 0.179 |
| ${KNN}_{D}$ | 0.290 ± 0.047 | 0.819 ± 0.051 | 0.838 ± 0.042 | 0.080 ± 0.096 | 0.545 ± 0.300 | 0.545 ± 0.293 |
| ${KNN}_{H}$ | 0.232 ± 0.062 | 0.767 ± 0.100 | 0.781 ± 0.091 | 0.082 ± 0.057 | 0.451 ± 0.302 | 0.635 ± 0.294 |
| ${KNN}_{E}$ | 0.422 ± 0.092 | **0.882 ± 0.041** | 0.898 ± 0.039 | **0.102 ± 0.025** | 0.588 ± 0.117 | **0.685 ± 0.112** |
| ${KNN}_{EH}$ | **0.478 ± 0.127** | 0.860 ± 0.047 | **0.916 ± 0.042** | 0.093 ± 0.019 | **0.624 ± 0.140** | 0.641 ± 0.117 |
| ${KNN}_{ED}$ | 0.474 ± 0.116 | 0.872 ± 0.048 | 0.914 ± 0.044 | 0.097 ± 0.025 | 0.602 ± 0.145 | 0.665 ± 0.131 |
| MIMIC-III dataset (At discharge) | | | | | | |
| ${KNN}_{Eucli}$ | 0.178 ± 0.027 | 0.694 ± 0.085 | 0.702 ± 0.075 | 0.233 ± 0.020 | 0.624 ± 0.149 | 0.519 ± 0.148 |
| LR | 0.222 ± 0.035 | 0.712 ± 0.067 | 0.769 ± 0.057 | **0.249 ± 0.026** | 0.607 ± 0.121 | **0.571 ± 0.124** |
| RF | 0.242 ± 0.032 | 0.740 ± 0.050 | 0.787 ± 0.043 | 0.229 ± 0.019 | 0.610 ± 0.131 | 0.523 ± 0.132 |
| LSTM | 0.242 ± 0.043 | 0.702 ± 0.063 | 0.794 ± 0.055 | 0.239 ± 0.021 | 0.612 ± 0.125 | 0.548 ± 0.123 |
| RNN | 0.221 ± 0.037 | 0.749 ± 0.061 | 0.755 ± 0.058 | 0.236 ± 0.018 | 0.620 ± 0.118 | 0.535 ± 0.119 |
| ${KNN}_{D}$ | 0.259 ± 0.053 | 0.701 ± 0.072 | **0.809 ± 0.063** | 0.228 ± 0.026 | 0.573 ± 0.169 | 0.542 ± 0.170 |
| ${KNN}_{H}$ | 0.198 ± 0.041 | 0.625 ± 0.106 | 0.756 ± 0.089 | 0.214 ± 0.026 | 0.582 ± 0.212 | 0.495 ± 0.212 |
| ${KNN}_{E}$ | 0.255 ± 0.038 | **0.824 ± 0.054** | 0.776 ± 0.055 | 0.247 ± 0.025 | 0.644 ± 0.128 | 0.541 ± 0.126 |
| ${KNN}_{EH}$ | **0.268 ± 0.044** | 0.818 ± 0.056 | 0.792 ± 0.051 | 0.244 ± 0.023 | 0.654 ± 0.125 | 0.530 ± 0.123 |
| ${KNN}_{ED}$ | 0.274 ± 0.050 | 0.813 ± 0.054 | 0.797 ± 0.057 | 0.243 ± 0.024 | **0.666 ± 0.131** | 0.518 ± 0.128 |
